# Orthotopic equine study confirms the pivotal importance of structural reinforcement over the pre‐culture of cartilage implants

**DOI:** 10.1002/btm2.10614

**Published:** 2023-10-19

**Authors:** Mylène de Ruijter, Paweena Diloksumpan, Inge Dokter, Harold Brommer, Ineke H. Smit, Riccardo Levato, P. René van Weeren, Miguel Castilho, Jos Malda

**Affiliations:** ^1^ Department of Orthopaedics, RMCU Utrecht, UMC Utrecht University of Utrecht Utrecht The Netherlands; ^2^ Department of Clinical Sciences, Faculty of Veterinary Medicine Utrecht University Utrecht The Netherlands; ^3^ Department of Biomedical Engineering Eindhoven University of Technology Eindhoven The Netherlands

**Keywords:** biofabrication, in vivo evaluation, melt electrowriting, multi‐technology, osteochondral

## Abstract

In articular cartilage (AC), the collagen arcades provide the tissue with its extraordinary mechanical properties. As these structures cannot be restored once damaged, functional restoration of AC defects remains a major challenge. We report that the use of a converged bioprinted, osteochondral implant, based on a gelatin methacryloyl cartilage phase, reinforced with precisely patterned melt electrowritten polycaprolactone micrometer‐scale fibers in a zonal fashion, inspired by native collagen architecture, can provide long‐term mechanically stable neo‐tissue in an orthotopic large animal model. The design of this novel implant was achieved via state‐of‐the‐art converging of extrusion‐based ceramic printing, melt electrowriting, and extrusion‐based bioprinting. Interestingly, the cell‐free implants, used as a control in this study, showed abundant cell ingrowth and similar favorable results as the cell‐containing implants. Our findings underscore the hypothesis that mechanical stability is more determining for the successful survival of the implant than the presence of cells and pre‐cultured extracellular matrix. This observation is of great translational importance and highlights the aptness of advanced 3D (bio)fabrication technologies for functional tissue restoration in the harsh articular joint mechanical environment.

**Translational Impact Statement**

In this study, a high resolution, hierarchical, mechanical stable framework that supports tissue regeneration is developed by converging biofabrication technologies. Long term, challenging in vivo evaluation demonstrates that the mechanical structure is more determining for the success of osteochondral implants than the presence of pre‐cultured cells. This observation is of great fundamental, as well as translational importance and supports the hypothesis that functional mimicking of the collagen architecture in the implants may be pivotal for optimal functionality and tissue restoration in vivo.

## 1 INTRODUCTION

The biomechanical function of the tissues within the skeletal system is pivotal to provide structure and strength to the body. Articular cartilage (AC) sustains similar forces as the skeletal bones, but also mitigates these by its shock‐absorbing character. AC is mechanically characterized by a combination of resilience and high resistance against compression and shear forces. This mechanical performance is permitted by the composition and structure of the extracellular matrix (ECM) of this relatively homogeneous, avascular and aneural tissue, combined with the strong interconnection of cartilage and bone into a cohesive functional structure (the osteochondral unit), that ensures load transmission and provides frictionless movement. The ECM of AC is a strong combination of a type II collagen network that is under intrinsic tension from highly hydrophilic proteoglycan aggregates.^1^, ^2^ The tissue is sparsely populated with cells (1%–12%), that only have a limited capacity of restoration of the tissue structure when skeletal growth has ceased, as the turnover of the main structural element, the collagen, is virtually nil in mature individuals.^3^, ^4^, ^5^ The calcified cartilage connects the AC with the mechanically widely different, much more rigid, subchondral bone. The resulting osteochondral unit allows, when in good health, proper joint function and nearly frictionless movement between opposing long bones.

In the quest for a regenerative solution for the unmet clinical need for the treatment of AC damage^6^, ^7^ several biomaterial‐based approaches have been explored, many of which involve the use of hydrogels for the cell‐friendly environment they can provide.^8^, ^9^, ^10^, ^11^, ^12^ Despite promising in vitro and small animal model results,^13^ these attempts did thus far not succeed to create a mechanically stable tissue that repeatably has stood the test of in vivo testing in a large animal model.^14^, ^15^, ^16^ Therefore, calls have been made to take a different approach in this area—from one that is primarily focused on optimizing the cell environment, towards that of recreating, more closely, the structural and mechanical features that define cartilage.^17^

**FIGURE 1:**
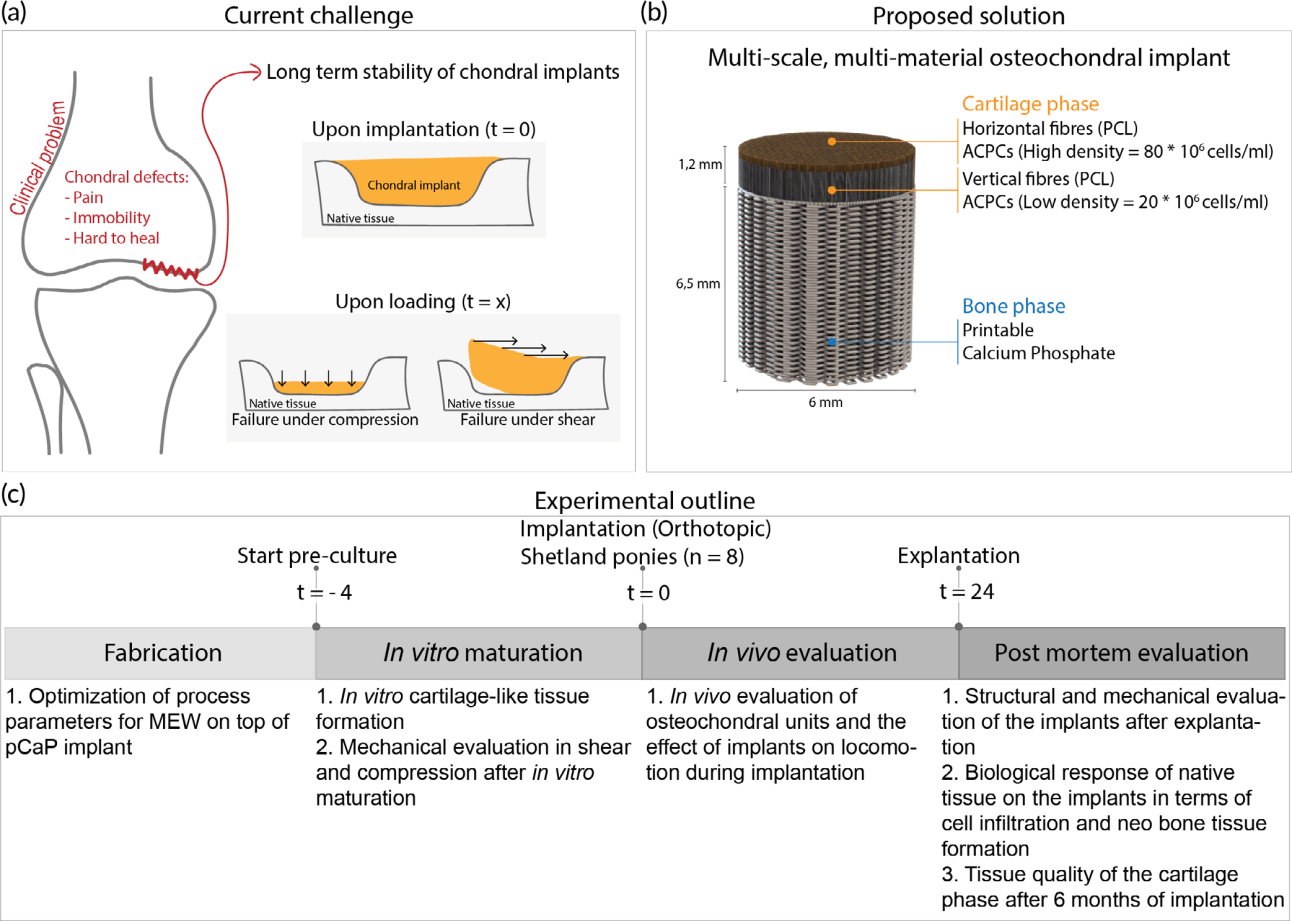
Schematic overview of this study. (a) Current regenerative implants for cartilage defects are not satisfactory, as these implants are not stable upon loading after orthotopic implantation. (b) Design details of the proposed multi‐scale, multi‐material osteochondral implant with details in the bi‐layered cartilage phase and regenerative pCaP bone phase. (c) Experimental outline of the performed study. ACPCs, articular cartilage‐resident chondroprogenitor cells. PCL, Polycaprolactone.

With the goal of restoring a biomechanically competent environment, this study (Figure 1) presents a function‐driven strategy by which an osteochondral implant was engineered based on the convergence of melt electrowriting (MEW)^18^, ^19^ with extrusion‐based 3D bioprinting within a single‐fabrication platform.^20^, ^21^ The implant was composed of a 3D‐printed calcium phosphate‐based (pCaP) bone phase, which was anchored with the cartilage phase through embedded polycaprolactone (PCL) fibers generated with MEW to securely connect the cartilage and bone components of the osteochondral unit.^22^ In addition, reinforcement of hydrogel structures with highly organized structures of these (sub)micrometer‐scale fibers increases the compressive and shear properties of hydrogel‐thermoplastic composites to values approaching those of the native cartilage tissue.^23^, ^24^, ^25^, ^26^ Moreover, the choice of relatively slowly degrading PCL as structure‐giving material may well guarantee the long‐term retention of the mechanical properties of the implant. Given the fact that the ECM is a major determinant of the biomechanical functionality and in view of the significant economic and regulatory challenges associated with the clinical translation of cell‐based regenerative techniques, we chose to compare a cell‐seeded with a cell‐free implant. For this, the most challenging large animal model,^27^ the horse, was used.

## 2 METHODS

### 2.1 Cell isolation, expansion, and differentiation

Equine articular cartilage‐resident chondroprogenitor cells (ACPCs) were isolated from healthy metacarpophalangeal joints of skeletally mature equine donors, as previously described.^28^, ^29^ These donors had been donated to science by their owners and procedures were followed according to the guidelines of the Ethical and Animal Welfare body of Utrecht University.

ACPCs were cultured in expansion medium until passage 5, after which they were cultured in chondrogenic differentiation medium (1 mL per implant) for 28 days. Expansion medium consisted of Dulbecco's modified eagle medium (31966, Thermo Fisher Scientific, USA) supplemented with 10% fetal bovine serum (Gibco, Thermo Fisher Scientific, USA), 1% penicillin–streptomycin (Gibco, Thermo Fisher Scientific, USA), 1% l‐ascorbic acid‐2‐phosphate (0.2 × 10^3^ M, Sigma Aldrich, USA), 1% non‐essential amino acids (100×, Gibco, Thermo Fisher Scientific, USA), and 5 ng/mL bFGF (Prepotech, UK), and medium was refreshed twice per week. Chondrogenic differentiation medium consisted of Dulbecco's modified eagle medium (31966, Thermo Fisher Scientific, USA) supplemented with 1% penicillin/streptomycin, 1% l‐ascorbic acid‐2‐phosphate, 1% ITS + Premix Universal culture supplement (Corning, USA), 2.5% HEPES (1M, Gibco, Thermo Fisher Scientific, USA), 0.4% dexamethasone (0.1 × 10^−6^ M, Sigma Aldrich, USA) and 0.1% recombinant human transforming growth factor‐β1 (TGF‐β1) (10 ng/mL, Prepotech, UK). Medium was refreshed three times per week. All cultures were performed under sterile and normoxic culture conditions at a temperature of 37°C and 5% CO_2_.

### 2.2 Materials

#### 2.2.1 Bioink

Gelatin methacryloyl (gelMA, degree of functionalization = 80%) was synthesized from low endotoxin gelatin (beMatrix gelatin LS‐H, type B, porcine skin, 300 Bloom, Nitta Gelatin, USA) as previously described.^30^, ^31^ Dialysis was performed for 4 days at 4°C, after which gelMA was lyophilized, and stored at –20°C until further use. Upon use, freeze‐dried gelMA was dissolved in PBS at 8% w/v. 2‐hydroxy‐1‐[4‐(2‐hydroxyethoxy)phenyl]‐2‐methyl‐1‐propanone (Irgacure 2959, BASF, Germany) was used as a crosslinking agent at 0.1% w/v and UV‐crosslinked for 15 min (UVP CL‐1000 Ultraviolet Crosslinker, 120,000 μJ/cm^2^). Gels were prepared at 8% w/v to match the same compressive properties observed in previous studies when using gelatin from different sources (Supplementary Figure 4).

#### 2.2.2 Printable calcium phosphate

The paste was prepared as a mixture of 2.2 g/mL of alpha‐tricalcium phosphate (α‐TCP) (average particle size = 3.83 μm, Cambioceramics, The Netherlands), 0.13 g/mL of nano‐hydroxyapatite (nano‐HA, particle size <200 nm, Ca5(OH)(PO4)3, Sigma‐Aldrich, USA) in a 40% w/v poloxamer‐solution (Pluronic® F‐127, Sigma‐Aldrich, USA). After scaffold fabrication, pCaP‐scaffolds were allowed to set for 4 days at 37°C under saturated humidity.

#### 2.2.3 Polycaprolactone

Medical‐grade polycaprolactone (PCL) (PURASORB PC 12, Corbion, The Netherlands) was used as received for the MEW process.

### 2.3 Implant design and fabrication

An osteochondral implant (Figure 1b, total height = 7.7 mm, diameter = 6 mm), consisting of three different layers, was fabricated by combining extrusion‐based printing with MEW and 3D bioprinting (3DDiscovery Evolution, regenHU, Switzerland). The bone compartment (height = 6.5 mm) of the implant consisted of printable calcium phosphate. This biomimetic bone compartment was fabricated from pCaP paste, by using pneumatic extrusion‐based 3D printing (3DDiscovery, regenHU, Switzerland). PCaP was printed on top of 50 layers (total height = 400 μm) of PCL MEW fibers to increase the interfacial strength between the bone and cartilage layer. PCaP cylindrical structures (diameter = 6 mm) were printed consisting of two non‐macropored layers where PCaP integrated with PCL micro‐fibers. Subsequently, macro‐pored layers were added by depositing PCaP strands (diameter = 250 μm) with a designed strand‐to‐strand distance of 700 μm in a double alternating pattern (orientation = 0°–0°–90°–90°). PCaP scaffold fabrication was performed at room temperature (20–25°C) with an extrusion pressure of 0.2 MPa and a translational speed of 2 mm/s. The cartilage compartment of the implant was bi‐layered with a distinction between the middle and deep zone and the superficial tangential zone as to provide a low‐volume percentage mechanical reinforcing framework that distributes the loads from the horizontally aligned fibers (superficial zone) to the cross‐sections of the box‐like structure (deep‐ and middle zone). The middle and deep zone (height = 1 mm) consisted of box‐like (laydown pattern 0°–90°–0°–90°) MEW PCL fibers (inter‐fiber distance = 300 μm), infused with 8% gelMA and ACPCs (20 × 10^6^ ml^–1^). The superficial tangential zone (height = 200 μm) consisted of MEW fibers (inter fiber distance = 100 μm) that were deposited in laydown pattern 0°–45°–90°–135°, with a slight offset to induce a higher density of tangentially aligned fibers. These fiber‐meshes were infused with 8% gelMA and ACPCs (80 × 10^6^ ml^–1^). As the in vitro tests focused on cartilage‐like tissue formation, the design of the scaffolds excluded the pCaP bone phase. To understand the effect of the bilayerd design (superficial, deep‐ and middle zone) as compared to conventionally used box‐shaped patterns, the box‐like structure (deep‐and middle zone) was added as a separate group for comparison in the histological, biochemical, and mechanical evaluation.

### 2.4 MEW fiber deposition optimization

The driving force behind MEW fiber deposition is the strong electrical field between the spinneret and the collector plate. By introducing a structure into this field, the electrical field is altered and therewith the fiber deposition is different. To decrease alteration of the fiber deposition on the implant due to this effect, a more stable electrical field around the edges of the implants was established by using an aluminum block to surround the pCaP bone phase (Supplementary Figure 3). To optimize MEW printing parameters, the measured distance between the MEW fibers (inter‐fiber distance) was compared with the programmed inter fiber distance, while using voltages ranging from 5 to 10 kV and relative collector distances (CDs) ranging from 5 to 9 mm. Additionally, the inter fiber distance on top of the pCaP implant was compared with the inter‐fiber distance onto the aluminum block. Pressure and collector velocity remained at 1.25 bar and 15 mm/s, respectively. Light microscopy (Olympus BX51, Olympus Nederland B.V., The Netherlands) was used to assess the fiber deposition quality, images were taken (Olympus DP73, Olympus Nederland B.V., The Netherlands) and measurements were performed with ImageJ (version 2.0.0‐rc‐54/1.51h).

### 2.5 SEM imaging

Scanning electron microscopy (SEM) (Phenom Pro Desktop SEM, Thermo Fischer Scientific, USA) was performed with an accelerating voltage of 10 kV to image the MEW fibers on top of the pCaP implant. Prior to imaging, samples were coated with 2 nm of gold to improve imaging quality.

### 2.6 Biochemical evaluation of 3D fabricated implants

To quantify the amount of sulphated glycosaminoglycans (GAGs) and correct them for DNA content, colorimetric dimethylmethylene blue (DMMB, Sigma Aldrich, USA) and fluorometric Picogreen (Quant‐iT‐Picogreen‐dsDNA‐kit, Invitrogen, USA) assays were performed, respectively. These assays were performed during in vitro culture (*t* = 1, 14, 28 days, as well as post‐mortem. Prior to these assays, implants were enzymatically digested overnight at 60°C using a papain digestion solution.

### 2.7 Immunohistological evaluation

Histological evaluation of the pre‐cultured constructs was performed to assess the distribution of cartilage‐like matrix components. The constructs were formalin‐fixed and embedded in paraffin. The in vivo explants were decalcified with EDTA for 6 months, prior to embedding in paraffin. EDTA was refreshed weekly and decalcification progress was checked weekly with micro‐CT imaging. Tissue sections (thickness = 5 μm) were deparaffinized with xylene and were rehydrated by gradual ethanol steps (100%–70%) prior to staining. Safranin‐O staining was used to visualize GAG distribution, combined with fast green (Sigma Aldrich, USA) to stain fibrous tissue, and hematoxylin (Sigma Aldrich, USA) to stain cell nuclei. A hematoxylin/eosin (H&E) staining was performed to provide an overview of matrix formation and implant stability.

Immunohistochemistry was performed to visualize type II collagen deposition. First, pronase (1 mg/mL, Roche, USA) and hyaluronidase (10 mg/mL, H2126, Sigma Aldrich, USA) were used for antigen retrieval, and sections were blocked with bovine serum albumin prior to primary antibody incubation II‐II6B3 (DSHB, USA). IgG was used as negative control staining. Samples were incubated over night at 4°C, washed, incubated with matching secondary antibody (1:100, IgG HRP, P0447) for 1 h at room temperature, and washed again. Subsequently, 3,3‐diaminobenzidine‐horseradish peroxidase (DAB, Sigma Aldrich, USA) was used to visualize the staining. After staining the cell nuclei with hematoxylin, pictures of histologically stained sections were taken with a light microscope (Olympus BX51, The Netherlands).

### 2.8 Mechanical analysis

The compressive modulus reported in this study reflects the observed behavior of the engineered constructs or native cartilage, instead of pure material properties. The compressive modulus and complex shear modulus of gel only constructs were compared with constructs that contained boxed reinforcement (reflecting the deep‐ and middle zone, a lattice pattern with fibers deposited in a laydown pattern 0°–90°–0°–90°, with a 300 μm strand‐to‐strand distance) and with constructs that contained bi‐layered reinforcement (with a superficial zone on top of the boxed reinforcement. The superficial zone includes alternating fibers deposited in a laydown pattern 0°–45°–90°–135°). The compressive modulus was evaluated at *t* = 0 days, after 14 days, and after 28 days. Compressive tests were performed on a Dynamical mechanical analysis (DMA, Q800, force range = 0.0001–18 N, TA instruments, USA). Compression modes included unconfined tests for engineered constructs before implantation and indentation for engineered constructs after explantation. Unconfined compression was performed by first applying a preload of 0.001 N to test samples and then strained to 30% at 20% strain/min. Indentation was performed on the engineered implant and adjacent native cartilage tissue by first applying a preload of 0.001 N, to ensure initial contact between the test samples and the flat indenter. During indentation with a cylindrical indenter (Ø = 2 mm), the cartilage was kept hydrated by continuously pipetting PBS over the surface of the implant. Engineered stress was calculated based on the force and specimen's unloaded cross‐sectional area, while engineered strain was based on ratio between unloaded specimen cartilage thickness (measured with caliper) and displacement of either unconfined compression platen or the indenter. For the indentation test, the loaded area was determined as the cross‐sectional area of the flat ended cylindrical indenter (Ø = 2 mm). The compressive Young's modulus was calculated from the slope of the stress strain curve within the elastic region by applying linear regression between 10% and 12% strain (of the cartilage depth).

The complex shear modulus was evaluated after 28 days of culture and measured with a rheometer (Discovery HR‐2, TA instruments, USA). An oscillatory rheometric protocol with plate‐plate (diameter = 25 mm) configuration was employed. After determining the viscoelastic (LVE) range with an amplitude sweep, a frequency sweep within this LVE range (0.05–500 rad/s, 0.01% strain) was performed under a 5% strain preload to prevent sliding of the sample. The complex shear modulus was calculated at 10 rad/s by dividing stress over strain.

### 2.9 In vivo evaluation of implants: the animal model

*Equus caballus ferus* (Shetland ponies, female, weight = 150–200 kg, *n* = 8, Table 1) was used as an animal model to evaluate the mechanical stability and regenerative capacity of the hierarchically structured osteochondral implants. Shetland ponies are considered a well‐established model for joint‐related diseases and no gender‐specific differences are shown in mature Shetland ponies with respect to joint‐related diseases or skeletal structure. As an internal control, a cell‐free osteochondral scaffold was used with the same architecture as the cell‐laden implants. Implants were designed to be implanted in a press‐fit approach with the bone anchor allowing for proper fixation.^32^ Implants were inserted in defects in the medial femoral ridge of the equine knee or stifle joint (Supplementary Figure 3B) under randomization (the key was made by a researcher who was not directly involved in this study) of implant placement in the left or right joint. Only after all data analysis was performed, the key was revealed to the involved researchers such that all analysis were performed blindly.

**TABLE 1:** Demographics of experimental animals used for this study.

Animal	Age (years)	Gender
1	6	Female
2	8	Female
3	12	Female
4	11	Female
5	6	Female
6	7	Female
7	5	Female
8	14	Female

The ponies arrived at the animal facility 4 weeks before starting the procedure to get acclimatized and were housed as a group at pasture. Prior to surgery they were moved to individual boxes and were fed a limited ration of concentrates with hay for maintenance and had free access to fresh water. All ponies were checked on “orthopedic soundness”, exclusion criteria were lameness, (intermittent) patella fixation, (partial) patellar luxation, effusion of the stifle, induction of lameness after distal limb and/or upper limb flexion tests, radiological abnormalities of the stifle.

Humane endpoints were defined as: development of septic arthritis, more than 4/5 lameness for 3 days duration during which the animal received maximal medication for pain relief (definition of different grades of lameness: 0 = not lame, 1 = stiff, but not lame as such, 2 = slightly lame 3 = clearly lame, 4 = seriously lame, 5 = on 3 legs (i.e., not willing to load the limb). Daily check‐ups were performed by professional caretakers.

For surgery, ponies were premedicated with detomidine (intravenous [IV], 10 μg/kg) and morphine (IV, 0.1 mg/kg) and anesthesia was induced with midazolam (IV, 0.06 mg/kg) and ketamine (IV, 2.2 mg/kg). Anesthesia was maintained with isoflurane in oxygen together with continuous rate infusion of detomidine (IV, 10 μg/kg/h) and ketamine (IV, 0.5 mg/kg/h). Meloxicam (IV, 0.6 mg/kg), morphine (Epidural injection, 0.1–0.2 mg/kg) and ampicillin (IV, 10–15 mg/kg) were administered pre‐operatively as analgesic medication and antibacterial preventative therapy, respectively.

The medial femoral ridge of the stifle joint was exposed by arthrotomy and an osteochondral lesion (diameter = 6 mm, depth = 7.2 mm) was surgically created using a power drill. The surgical area was flushed by saline for cooling and removal of debris. Cell‐laden constructs were implanted press‐fit in a randomly chosen hind limb, with the cell‐free control being implanted in the contralateral limb. After closing the arthrotomy wound in 4 layers in routine fashion, procaine penicillin was administered (Procapen, intramuscular [IM], 20 mg/kg). Post‐operatively, nonsteroidal anti‐inflammatory medication (meloxicam per os [PO], SID, 0.6 mg/kg) was administered for 5 days and opioids (tramadol, PO, BID, 5 mg/kg) was administered for 2 days.

Post‐operatively, the animals directly loaded the joints and were kept stabled for 6 weeks with daily monitoring of vital signs, lameness checks at walk and examination of the operated joints for swelling or other signs of inflammation. In weeks 5 and 6, they were hand‐walked for 10 min twice daily and from week 7 they were kept at pasture. Quantitative gait analysis and radiographic exams were performed at 3 weeks, 3 months, and 6 months post‐operatively. After 6 months, the animals were humanely euthanized by intravenous injection of an overdose of pentobarbital (IV, 1400 mg kg^−1^ body weight), following sedation (detomidine IV, 10 μg/kg) and induction (Midazolam [IV, 0.06 mg kg^−1^ body weight] and ketamine IV, [2.2 mg kg^−1^ body weight]). All procedures had been approved by the ethical and animal welfare body of Utrecht University (Approval no. AVD108002015307 WP23).

### 2.10 Gait analysis during in vivo testing period

During the acclimatization period, the ponies were trained on a treadmill (Mustang, Fahrwangen, Switzerland) using a standard protocol for treadmill habituation.^33^ A total of 28 spherical reflective markers (diameter = 24 mm [topline] and 19 mm [elsewhere]) were attached with double‐sided tape and second glue to anatomical landmarks (Supplementary Figure 2B). Kinematic data were collected at trot using six infrared optical motion capture cameras (ProReflex, Qualisys, Gothenburg, Sweden) recording for 30 s (frame rate = 200 Hz) at each session to obtain a sufficient number of strides.

To process the data, the reconstruction of three‐dimensional coordinates of each marker was automatically calculated by Q‐Track software (Qtrack, Qualisys, Gothenburg, Sweden). Each marker was identified and labeled using an automated model (AIM model) and manual tracking and raw data were exported to Matlab (version 2018a, Niantics, California) for further analysis. Using custom written scripts, two symmetry parameters were calculated using the vertical displacement of the head and pelvis (tubera sacrale) markers, for each stride. Additionally, the differences between the two vertical displacement minima of the head (MinDiff_head_) and pelvis (MinDiff_pelvis_) were calculated. Using the markers, limb‐segments were formed and angles between these limb‐segments were calculated. The difference between the maximal and minimal angle was defined as the range of motion (ROM) of a joint. For each timepoint, the mean value of all strides for each parameter was calculated.

### 2.11 Evaluation of in vivo neo bone tissue formation (μ‐CT)

Microcomputed tomography (μ‐CT) was employed for the quantitative analysis of the bone compartments from the harvested osteochondral lesions (*N* = 8 for cell‐laden constructs, *N* = 8 for cell‐free constructs). Six freshly made osteochondral grafts were scanned in a μ‐CT scanner (Quantum FX‐Perkin Elmer) to quantify the initial volume of pCaP material, pre‐operatively. The post‐mortem harvested tissue containing the defect area and the surrounding native tissue were similarly scanned (voltage = 90 kV, current = 200 μA, voxel size = 30 μm^3^ and total scanning time = 3 min). Subsequently, the 3D‐reconstructed images were processed and analyzed using image J.^34^ and Bone J.^35^ software. Two‐dimensional regions of interest (ROIs) were selected in an axial plane at the boundary between the defect and the surrounding native tissue and interpolated to form 3D‐volumes of interest (VOI). Thresholding was performed to separately selected area of ceramics and newly formed bone for further calculation. Thresholding values were selected based on the image histogram where different intensity of ceramic and newly formed bone can be identified. After thresholding, processed images were compared with original images. Then, the percentages of mineralized newly formed bone, of non‐mineralized tissue and of remaining ceramics, including the percentage of ceramics volume loss, were quantified.

### 2.12 Evaluation of in vivo cartilage formation

After explantation, the implants were macroscopically evaluated and pictures were taken with a stereomicroscope (Olympus stereomicroscope (Olympus Soft Imaging Solutions GmbH, The Netherlands). Biopsies (diameter = 1 mm) of the newly formed tissue and adjacent native tissue were taken for biochemistry. The rest of the explant was further processed for immuno(histological) evaluation.

### 2.13 Statistics

Data is presented as mean ± standard deviation, unless otherwise specified. All in vitro studies were performed in triplicate, mechanical analysis was performed with *n* = 5, and printing accuracy was assessed with *n* = 10. To analyze the micro‐CT data, Matlab (version R2018b, The MathWorks, Inc.) was used to perform Wilcoxon signed rank tests. To test statistical differences between groups, either an unpaired *t*‐test, or a one‐way ANOVA with post hoc Bonferroni test was performed. Difference between groups was considered statistically significant if *p* < 0.05. For the in vivo study, randomization was done, by a researcher that was not involved in this study, to decide which construct (cell‐seeded or not) was implanted in which stifle joint and post‐explantation evaluation was performed blindly by making use of a key that was only revealed after all data analysis was performed.

## 3 RESULTS AND DISCUSSION

**FIGURE 2:**
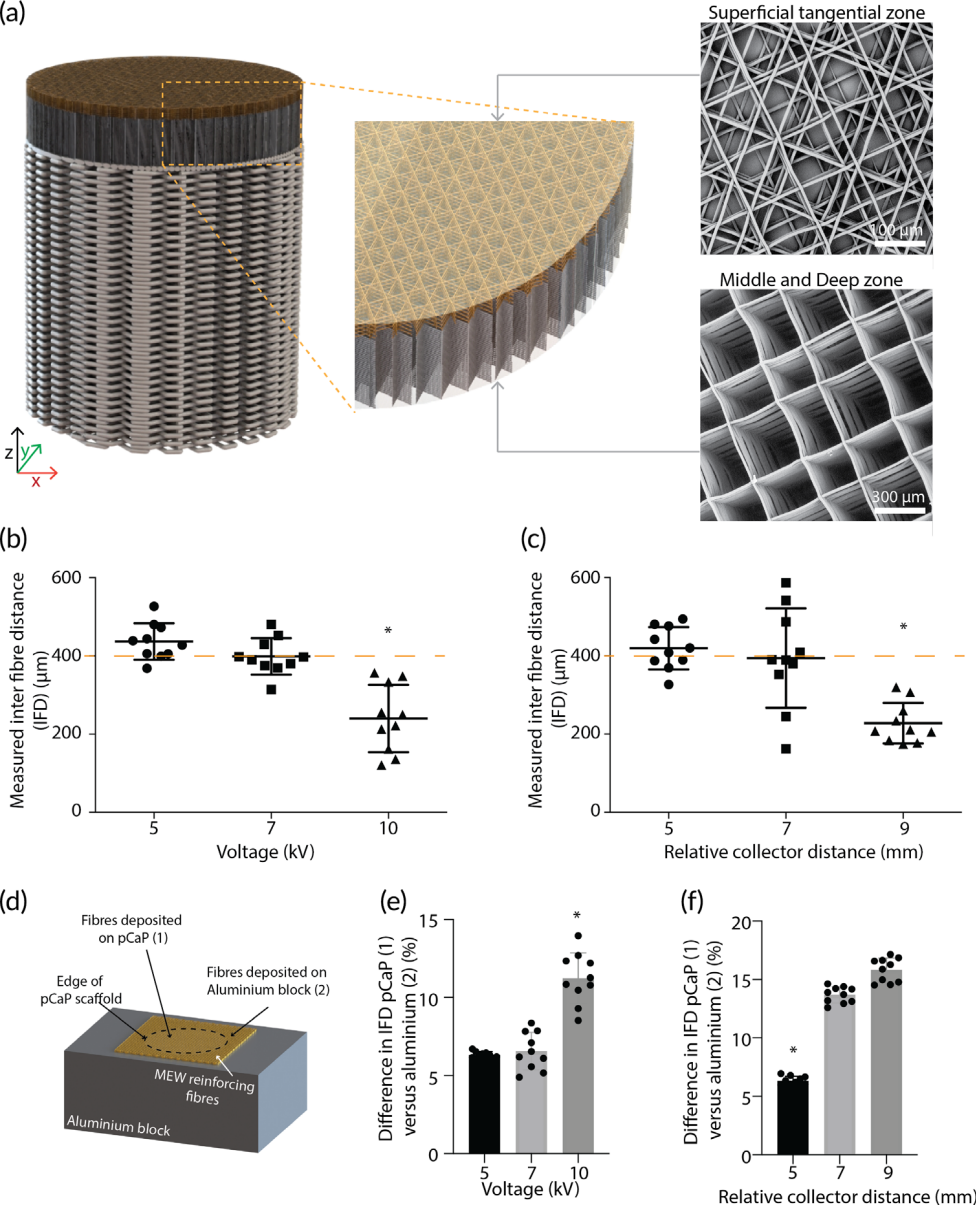
Optimization of process parameters for deposition of reinforcing microfibers on top of the pCaP implant. (a) Illustration of bi‐layered cartilage phase, including SEM images that confirm the directionality of the fibrous component of the superficial tangential zone, and the middle and deep zone. (b, c) Measured inter‐fiber distance is affected by both voltage and relative collector distance (*n* = 10). (d) Schematic indication of measurements taken (e, f) Effect of voltage and relative collector distance on difference of inter‐fiber distance on top of the pCaP bone phase as compared to on top of the aluminum block. Higher % reflects more piling up of reinforcing fibers at the edges of the pCaP bone phase (*n* = 10). Error bars represent standard deviation, “*” Statistically different from all other groups (*p* < 0.05).

The potential of biofabrication technologies for the regeneration of musculoskeletal tissues has been postulated for over a decade, but long‐term functionality and mechanical stability had not yet been reported within large animal models.^36^, ^37^, ^38^, ^39^ The convergence of technologies shows great potential for the fabrication of functional tissue constructs. Here, the design of the osteochondral implants was inspired by native tissue, and specifically the type II collagen fiber structure. To realize this design, hierarchy in fiber orientation and cell density was successfully achieved by converging extrusion‐based bioprinting and MEW processes. Patterning of MEW fibers onto the non‐conductive CaP bone phase was obtained by ensuring a constant electrical field strength and an electrical force perpendicular to the substrate; an aluminum block surrounded the pCaP plug during fabrication as to prevent fibers piling up at the edges of the pCaP plug. Control over fiber deposition on top of the pCaP plug allowed for fabrication of a bi‐layered cartilage phase representing the middle/deep and superficial tangential zones of the native tissue (Figure 2a). While MEW a uniform box‐structure, an increase in voltage resulted in a decrease in the strand‐to‐strand distance between the deposited MEW fibers (inter‐fiber distance) on top of the pCaP bone phase of the osteochondral implant (Figure 2b). Furthermore, an increase in relative CD resulted in additional decrease in the measured inter‐fiber distance (Figure 2c). A voltage of 7 kV and a relative CD of 5 mm resulted in more accurate inter‐fiber distance (409.1 ± 49.0 μm) as compared to the programmed value (400 μm). Additionally, this combination of parameters led to the most homogeneous fiber deposition, as no fibers were piling up at the edges of the pCaP scaffold; the difference between the inter‐fiber distance on top of the pCaP plug and onto the aluminum block was neglectable (Figure 2d–f).

Using a voltage of 7 kV and a relative CD of 5 mm a bi‐layered cartilage phase (Figure 2a) with a clearly distinct pattern in the layer representing the middle and deep zones compared to the layer representing the superficial tangential zone was obtained. The middle and deep zones demonstrated a uniform box structure and “z‐directional” stacking (according to local coordinate axis in Figure 2), whereas the superficial tangential zone featured primarily tangentially oriented fibers with little z‐directional stacking. For this superficial tangential layer, programmed inter‐fiber distances of 100, 200, and 400 μm in a laydown pattern of 0°–45°–90°–135°–180° corresponded with interconnected pores that showed an average inter‐fiber distance of 49.2 ± 6.2 μm, 110.1 ± 17.4 μm, and 359.2 ± 29.6 μm, respectively (Supplementary Figure 1A, B). Cells were able to, partially, penetrate all meshes, irrespective of pore size (Supplementary Figure 1C); however, most cells were caught by meshes that were fabricated with an inter‐fiber distance of 100 μm (Supplementary Figure 1D). Therefore, 100 μm inter‐fiber distance, which was the smallest that resulted in the creation of a smooth surface, was selected for the superficial tangential zone of the osteochondral implants.

The fiber design of the bi‐layered cartilage phase was inspired by the structurally important collagen type II arcs, which are aligned parallel to the surface at the superficial zone and normal to the surface at the deep and middle zone.^40^ In line with native tissue, it was shown that MEW fibers that were aligned parallel to the surface allow for improved load distribution,^25^ whereas the cross‐sections of the fibers in a boxed‐structure mainly withstand the compressive loading.^41^ Even though fiber diameters obtained with the MEW process are already one to two orders of magnitude smaller than those produced with conventional extrusion‐based techniques, they are still much thicker than the native collagen fibers.^42^, ^43^ Yet, the aim of this study was not to fully recapitulate the structure of native collagen fibers but rather the mechanical function of such. As a technique, MEW has unique properties to fulfill this aim, even further exploited by converging with other bioprinting technologies. The convergence of MEW with extrusion based bioprinting of ceramic and hydrogel allowed for osteochondral implants that are unique in the complexity and biomimicry. This high level of complexity and biomimicry was only possible as of the high resolution, high reproducibility, and small fiber diameters (micrometer scale) of the MEW fibers. This not only allows for a unique freedom in design of the bi‐layered cartilage zone, but also for ample space for the cells to migrate into the scaffold and to produce tissue‐specific matrix. Furthermore, the combination of the material choice and small diameter of the reinforcing fibers is hypothesized to still allow for a mechanoenvironment similar to native tissue, where other reinforcing strategies, such as the ones that use FDM fibers, potentially risk a stress‐shielding effect on the cells.^23^

**FIGURE 3:**
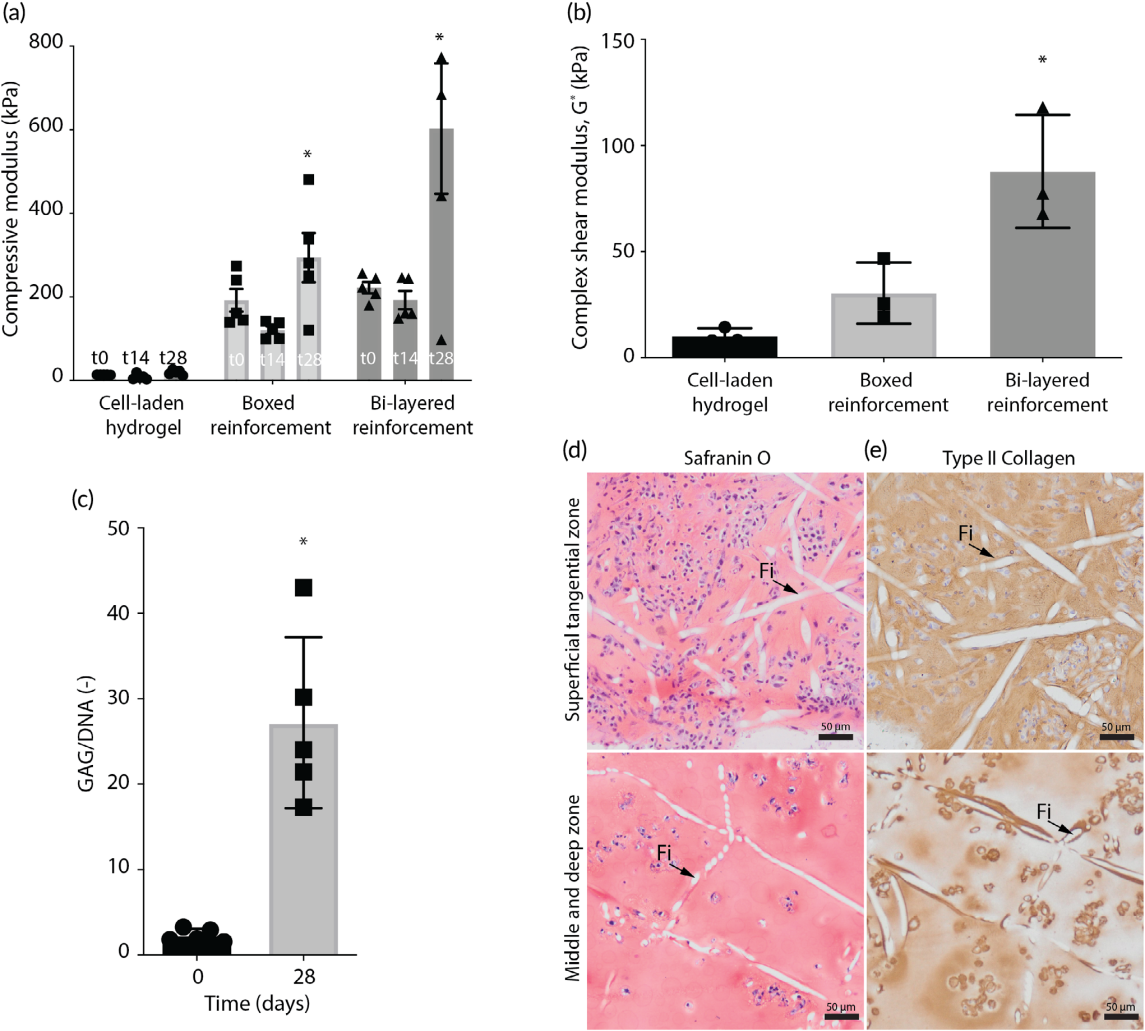
Mechanical analysis and in vitro cartilage‐like tissue formation of the osteochondral implants. (a) A general increase in compressive modulus over time was found and bi‐layered reinforcement has increased the compressive modulus after 0, 14, and 28 days of culture (*n* = 3). (b) Bi‐layered reinforcement has increased the complex shear modulus after 28 days of culture (*n* = 3). (c) 28 days of in vitro culture resulted in an increase in quantitative GAG deposition, normalized per DNA (*n* = 3). (d) Homogeneous distribution of safranin‐O and type II collagen staining was found in both the superficial tangential, and middle and deep zone after 28 days of in vitro culture, top view (Fi = MEW fiber). Error bars represent standard deviation, *p < 0.05, one‐way ANOVA, post hoc Bonferroni (a, b), unpaired *t*‐test (c).

After 28 days of in vitro culture, the bulk compressive modulus of the bi‐layered reinforced constructs was significantly higher (603.2 ± 205.4 kPa) than those of the boxed‐reinforced (294.2 ± 147.5 kPa) and non‐reinforced cell‐laden hydrogels (19.6 ± 5.8 kPa). For all cell‐laden groups these values were higher as compared to the compressive modulus prior to in vitro culture when the compressive modulus of the cell‐laden hydrogel was 13.9 ± 0.2 kPa, improved by the uniform boxed‐reinforcing fiber structure to 192.3 ± 54.6 kPa and even further improved with the bi‐layered fiber structures to 222.6 ± 30.7 kPa (Figure 3a). Notably, the inclusion of the bi‐layered reinforcing structure resulted in a higher complex shear modulus (87.8 ± 21.7 kPa) after the in vitro culture compared to the non‐reinforced cell‐laden hydrogel (10.3 ± 3.0 kPa) and the boxed‐reinforced constructs (30.5 ± 11.8 kPa) (Figure 3b). It is important to note that the mechanical properties reflected here, concern the bulk properties and that our hybrid scaffold design decouples the load bearing capacity of the scaffold (provided by the micrometer scale fibers) from the biologically favorable environment for the cells (provided by the hydrogel).

The mechanical properties of hydrogel scaffolds that are reinforced (e.g., with MEW micrometer scale fibers) depend on multiple factors, including the materials used and the architectures chosen. This allows for a wide range in mechanical properties and the mechanical properties of the pre‐cultured constructs in this study seem promising. The hierarchy in the fiber orientation, that is, a uniform boxed structure representing the middle/deep zones and a zone with primarily tangentially‐oriented fibers to represent the superficial tangential zone, resulted in increased compressive and shear properties, as well as improved load distribution.^25^, ^41^ In fact, the inclusion of such a thin layer of tightly packed and tangentially oriented fibers at the implant surface, has recently been shown to enable the axial loads to be distributed over a larger volume of the underlying middle and deep reinforcing region, therefore more effectively transferring axial loads throughout the engineered construct.^25^ Furthermore, the convergence of the technologies (extrusion based ceramic printing and MEW of the reinforcing fibers) previously resulted in a firm integration between the cartilage and bone components.^22^ This firm integration is also included in the unique design of the osteochondral implants in this study, that further include a closer biomimicry in the fibrous and cellular aspects of the cartilage phase.

The increase in mechanical properties during the pre‐culture period might be attributed to the increase of glycosaminoglycan (GAG) in the constructs. The in vitro pre‐culture period resulted in an average GAG content of 27.2 ± 9.8 μg GAG/μg DNA (Figure 3c), which was, together with type II collagen, homogeneously distributed throughout the middle/deep, and the superficial tangential zones of the cartilage component of the implant (Figure 3d). Further, the reinforcing MEW fibers, which appeared in the stained histological sections as white, where shown to preserve the original designed orientation established during the printing process (Figure 2a). Next to the reinforcing and load distributing effect of the fiber‐dense superficial layer, this layer might also play a role in the entrapment of deposited GAGs and swelling restriction, as is known for native collagen fibers.^43^

Pre‐cultured osteochondral constructs were implanted in the equine model in the medial femoral ridge of the stifle joint, slightly below the articulating surface (0.5 ± 0.4 mm). The equine model has shown to be a relevant model for orthotopic in vivo studies as equine joints are roughly similar in size (for ponies), have comparable cartilage thickness and biochemical composition of the cartilage, and show similar pathology as human patients for critical size defects.^5^, ^44^ Moreover, as the equine model is considered the most challenging model for load bearing, joint‐related studies,^27^ the translation to the human patient of the results regarding the mechanical stability of these osteochondral implants is promising. During surgery and post‐surgery recovery, no complications occurred and radiographic examination (x‐rays) confirmed the correct implant orientation of the implants after 3 and 6 months of implantation (Supplementary Figure 2A).

During the implantation period, gait analysis revealed that symmetry parameters were not affected by the type of implant, as no difference was found between the cell‐laden and cell‐free group at any time point (Supplementary Figure 2B–K). Symmetry parameters (MinDiff Head and MinDiff Pelvis) show a slight deviation after 3 months of implantation, yet these values were back to base level after 6 months of implantation (Supplementary Figure 2C, D). Both pelvis roll range of motion (ROM) and pelvis yaw ROM significantly increased within 3 months of implantation (Supplementary Figure 2E, F) and pelvis yaw showed a further increase until 6 months of implantation (Supplementary Figure 2F). Pelvis pitch ROM slightly decreased within the first 3 months of implantation (Supplementary Figure 2G). No differences in limb parameters (fetlock extension, limb height, protraction, and retraction) were found between the cell‐laden and cell‐free implants. (Supplementary Figure 2H–K).

**FIGURE 4:**
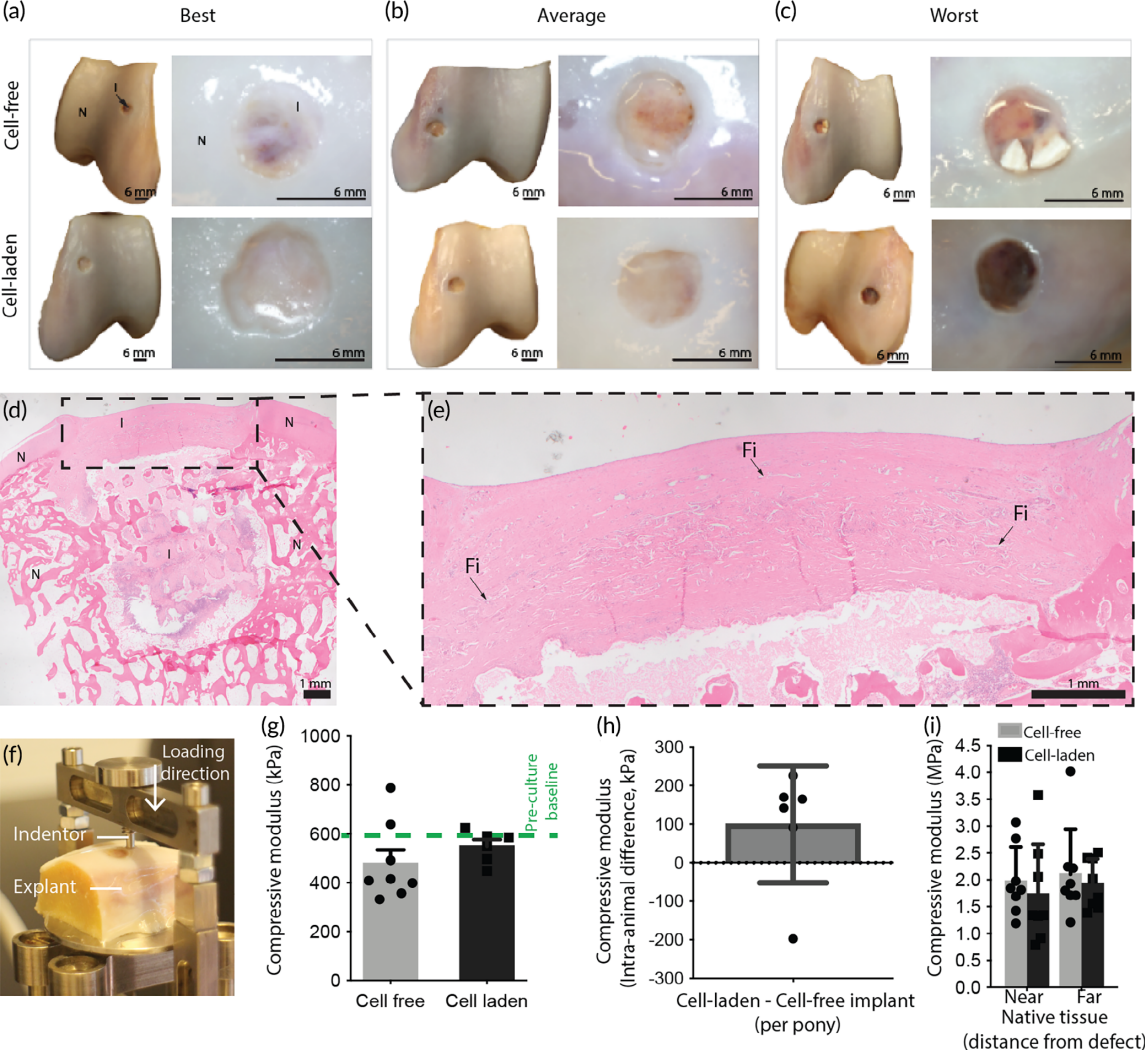
Structural and mechanical evaluation of the implants. (a–c) Macroscopic evaluation of the explants showing the best (a), average (b), and worst (c) samples for the cell‐free and cell‐laden implants. (d, e) H&E staining of a cell‐laden implant, highlighting the presence of the reinforcing fibers throughout the cartilage phase (*N* = native tissue, I = implant, Fi = MEW fiber). (f) Compressive mechanical testing of the implants and native tissue after explantation. (g) After 6 months of implantation, a similar compressive modulus was found for the implants, as compared to the pre‐implantation timepoint (*n* = 8 for cell‐free implants, *n* = 6 for cell‐laden implants as of inhomogeneous tissue formation on 2 samples). (h) Internal difference per pony between the cell‐laden and cell‐free implants (*n* = 6). (i). Compressive modulus of native tissue measured near the defect site and far from the defect site (*n* = 8). Error bars represent standard deviation, *p < 0.05, one‐way ANOVA, post hoc Bonferroni.

After 6 months of implantation, in most (13 out of 16) of the implants repair tissue was observed macroscopically (Figure 4a–c). At most of the sites that had received a cell‐free implant the defect was partially filled with repair tissue with a transparent to whitish color (Figure 4b). Out of all 8 initially cell‐free implants, 1 implant resembled the best outcome, 6 the average outcome, and 1 the worst outcome. The repair tissue in the defects treated with initially cell‐laden implants had a more whitish and less transparent character (6 out of 8) (Figure 4a–c). Out of all 8 initially cell‐laden implants, 2 implants resembled the best outcome, 4 the average outcome, and 2 the worst outcome. Cross‐sections of the implants, stained with Hematoxylin & Eosin (H&E), revealed lateral bone ingrowth into the osteal anchor of the implant (Figure 4d). Additionally, these tissue sections confirmed that the cartilage compartments of the implants still remained intact and provided a good filling of the original defect after 6 months of implantation (Figure 4d, e). Moreover, the reinforcing MEW fibers (“Fi” in Figure 4e) remained visible throughout the entire cartilage compartment of the implant.

Mechanical analysis under indentation loading (Figure 4f) showed no significant difference (*p* = 0.073) in compressive modulus between the cell‐free (0.5 ± 0.2 MPa) and cell‐laden implants (0.6 ± 0.1 MPa) (Figure 4f, g). Comparing the compressive properties after 6 months of implantation with those prior to implantation revealed that the compressive properties of the cell‐laden implants were conserved and no significant decrease in compressive modulus was found after 6 months implantation. Importantly, at the time of explantation, the initially cell‐free implants had gained significant additional compressive properties and comparison with internal controls revealed that there was no significant difference with the compressive modulus of the cell‐laden implants (Figure 4h). Additionally, composition of the native tissue near (<2 mm) and further away (>10 mm) from the treated defect site was independent of the presence or absence of cells in the implanted construct (Figure 4i). Although the implants did not match the compressive properties of native tissue yet, the mechanical stability to withstand the in vivo environment that was shown in this study, is of major importance. Further research can be done to recapitulate the biological mimicking of the matrix that can be deposited and remodeled by the cells, throughout the micro fibrous scaffold that prevents the implant from destruction. The implant that was developed in this study can be used as a scaffold for multiple emerging biomimicking or matrix stimulative strategies while providing joint functionality, especially since the results of the initially cell‐free and pre‐cultured cell‐laden implants showed similar results.

The work reported here is the first to underscore the postulation that better understanding of the mechanisms of collagen structure development combined with evolving (bio)fabrication and printing approaches would lead to further functional mimicking of native AC tissue.^13^ Achieving this mechanically stable, resorbable framework in a representative large animal model suggests that the technical solution to restore AC defects potentially lies in the convergence of (bio)printing technologies that enable creation of such a mechanical environment that supports ECM production in vivo.^20^, ^45^, ^46^ This structural framework is envisioned to be used with different biomaterials for tissue regeneration, as developments in biomaterial research continues and subsequently evolves tissue regeneration. Future studies that include this framework with other biomaterials should consider the degradation and migration potential of the biomaterials used, as these factors could significantly affect matrix deposition in vivo, especially for cell‐free implants, which do not include pre‐cultured cartilage‐like matrix components. Next to the use of different hydrogel systems within this polymeric framework, the framework itself could also be further engineered to stimulate chondrogenic differentiation and matrix production. For example, atmospheric‐pressure plasma treatment of PCL microfibers has shown to covalently bind TGF‐β1, which subsequently increases cartilage‐like matrix production in vitro.^47^

**FIGURE 5:**
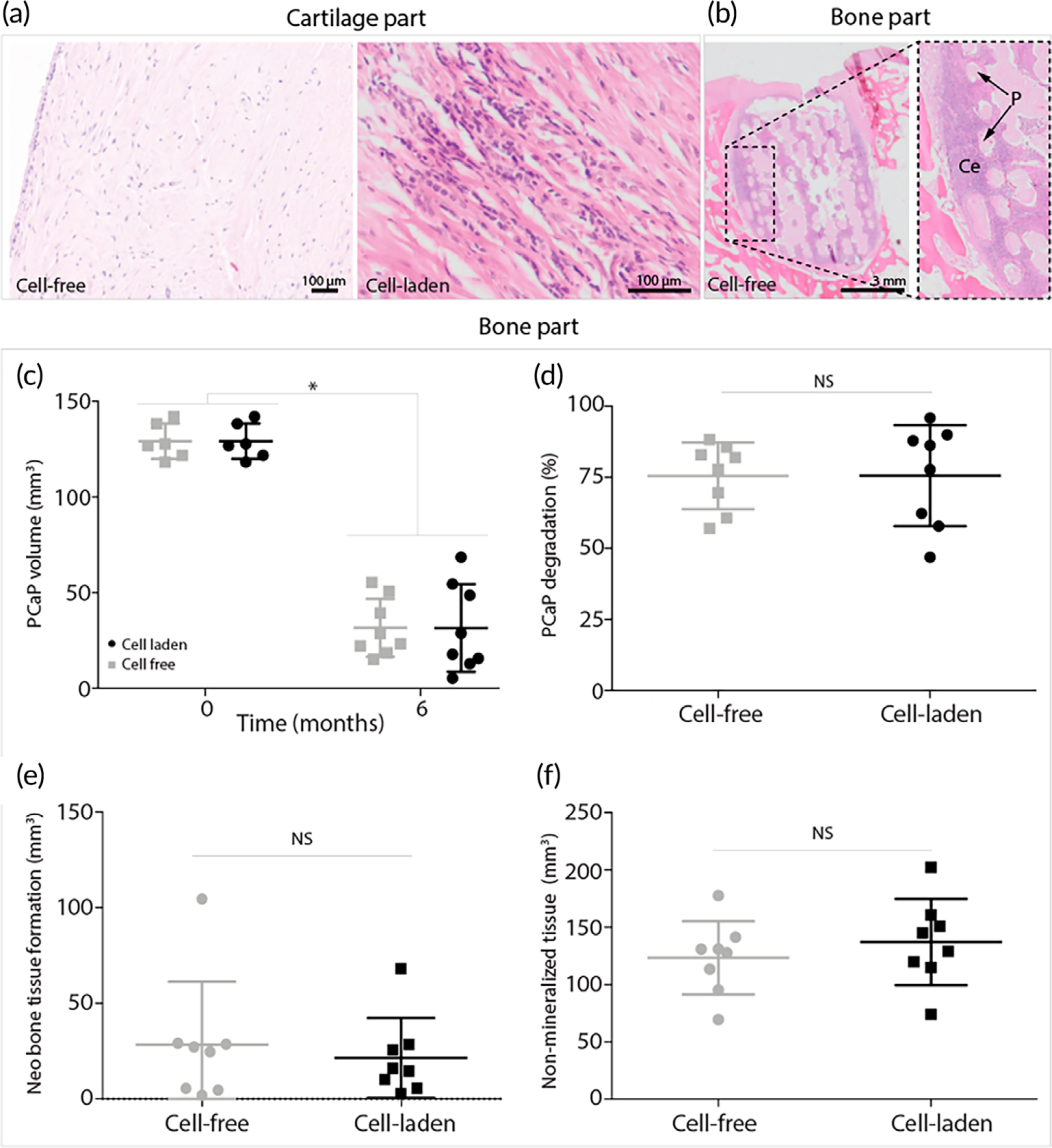
Interaction of native tissue and implants. Hematoxylin and eosin staining to assess cell infiltration in the cartilage and bone part and cell morphology in the cartilage part. (a) Cell infiltration in cartilage part of the cell‐free implant and cell morphology (mixture of spindle/fibrous and rounded chondrogenic morphology) in the cartilage part of the cell‐laden implant. (b) Cell infiltration and bone formation bone part of cell‐free implants. (P = pCaP voids, Ce = cells). (c–f) Quantification of micro‐CT data after 6 months of implantation (*n* = 8). (c) Total volume of pCaP over 6 months. (d) PCaP degradation over 6 months in percentages. (e) Volume of neo‐bone tissue formation. (f) Volume of non‐mineralized tissue. Error bars represent standard deviation, *p < 0.05, NS = no significant difference, unpaired *t*‐test.

Even though the main focus of this study was the generation of a mechanically stable osteochondral plug, in this study pCaP was used for tissue regeneration in the bone component, and gelMA for tissue regeneration in the cartilage component. Interestingly, after 6 months of implantation, a hematoxylin and eosin (H&E) staining of tissue sections showed abundant infiltration of cells in the cartilage compartment of the cell‐free implants (Figure 5a). Cells in the cartilage compartment of both the initially cell‐free and cell‐laden implants showed a mixed morphology of fibrous/spindle‐shaped and rounded cells (Figure 5a). From this study, no conclusions can be made on the origin of the infiltrated cells and future studies should be conducted to further understand complete regeneration of cartilage tissue. Yet, immunohistochemistry (Supplementary methods, Supplementary Figure 5) showed negative staining for collagen type X in the cell laden and cell free samples, suggesting that both the initially implanted as well as the infiltrated cells did not undergo hypertrophic differentiation. Although it is known that ACPCs are not prone to undergo hypertrophy in vitro^28^ it is promising that this was also not shown in this in vivo study. Furthermore, the bone compartment of all implants, based on osteoconductive ceramics,^48^ showed considerable cell infiltration, and all implants additionally showed neo‐bone tissue formation (Figure 5b).

Micro‐CT imaging revealed degradation of the pCaP bone anchor. The pCaP volume decreased significantly from 129.2 ± 8.5 mm^3^ to 31.7 ± 14.2 mm^3^ and 31.6 ± 21.5 mm^3^ for the cell‐free and cell‐laden implants, respectively (Figure 5c). No significant difference in percentage of pCaP degradation was found between the cell‐free (75.5 ± 11.0%) and cell‐laden (75.6 ± 16.6%) groups (Figure 5d). Although all implants showed bone infiltration into the pCaP part, some bone resorption around the pCaP part was also found. Neo‐bone tissue formation was found in both the cell‐free and cell‐laden implants (Figure 5e). Interestingly, this neo‐bone tissue formation was irrespective of the cartilage component, as there was no significant difference in volume of neo‐bone tissue formation between the cell‐free (28.3 ± 30.8 mm^3^) and cell‐laden (21.48 ± 19.5 mm^3^) implants (Figure 5e). Additionally, no significant difference was found for non‐mineralized tissue between the cell‐free (123.4 ± 30.0 mm^3^) and cell‐laden (137.1 ± 35.2 mm^3^) implants (Figure 5f).

**FIGURE 6:**
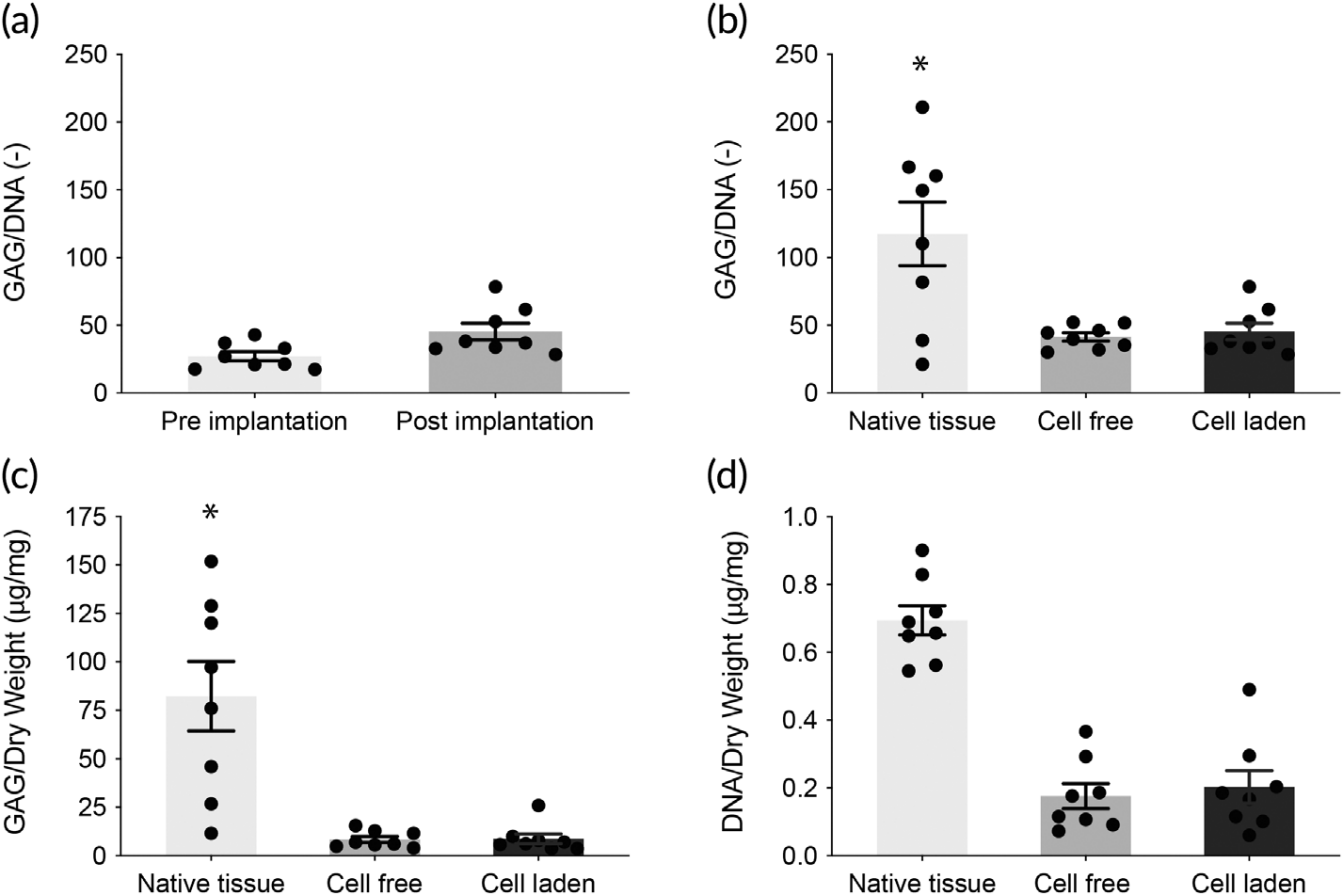
Tissue quality of the cartilage phase of the implants after 6 months of implantation. (a) Comparison of GAG/DNA before and after implantation of the cell‐laden, pre‐cultured implants (*n* = 8). (b) Quantitative biochemistry shows no difference between the cell‐laden and cell‐free implants in average GAG/DNA (*n* = 8). (c) Average GAG/dry weight compared with native tissue (*n* = 8). (d) Average DNA/dry weight compared with native tissue (*n* = 8). Error bars represent standard deviation, *p < 0.05, one‐way ANOVA, post hoc Bonferroni.

In the cartilage component, the implants showed an increased GAG/DNA content after 6 months of implantation compared to the pre‐culture (t28) timepoint (Figure 6a). Interestingly, within the initially GAG‐deprived cell‐free implants, a significant increase in GAG/DNA was found up to the level of the pre‐cultured cell‐laden samples, after 6 months of implantation (Figure 6b). Also, no significant difference in GAG/DNA was found (*p* = 0.1813) between these cell‐free (41.5 ± 9.0 μg/μg) and the cell‐laden implants (45.4 ± 16.3 μg/μg) (Figure 6b). Nevertheless, both implants showed significantly less GAG/DNA content in comparison to the surrounding native tissue (117.5 ± 74.4 μg/μg). A similar trend was observed for the overall GAG content of the implants normalized per dry weight. A significant increase in GAGs was shown for the initially GAG deprived cell‐free implants, and no significant differences were found between the cell‐free (8.7 ± 4.2 μg/mg) and cell‐laden implants (8.8 ± 6.8 μg/mg) (Figure 6c). Interestingly, also no difference in DNA content, normalized per dry weight, was found between the cell‐free (0.176 ± 0.105 μg/mg) and cell‐laden implants (0.203 ± 0.128 μg/mg) (Figure 6d).

An important observation is that the cell‐free gel‐fiber combination used as the cartilage phase of the implants attracted chondrogenic ECM producing cells in the in vivo situation and that GAG/DNA content of the neo‐tissue formed in cell‐free implants was equal to that seen in the cell‐laden, pre‐cultured implants. After 6 months of implantation, macroscopically all implants (cell‐free and cell‐laden) showed formation of a repair tissue. Biochemical assessment of the post‐mortem retrieved implants showed a further increase in GAG/DNA for the cell‐laden implants (45.4 μg/μg of GAG/DNA) compared to the pre‐implantation timepoint (28.0 μg/μg of GAG/DNA), providing evidence that additional ECM production occurred after in vivo implantation. This finding shows superior performance of these osteochondral plugs compared to earlier equine studies in which GAG content of pre‐cultured implants decreased, presumably due to leaking out of matrix components as a result of the exposure to loading.^32^, ^49^

## 4 CONCLUSION

This study demonstrates that implants with a defined structural hierarchy in the cartilage compartment, produced using converged fabrication technologies, can withstand the challenging in vivo situation in a large animal model for a prolonged time‐period. This convergence of biofabrication technologies allowed the manufacture of mechanically stable, resorbable implants with multi‐scale architectures, at a high resolution. The bi‐layered micro fiber reinforcement in the chondral compartment and its integration with the bone anchor, substantially improved the compressive and shear properties of the implant and is therefore structurally important. Based on the in vivo tissue formation, this study suggests that the mechanical structure is more determining for the success of osteochondral implants of this size than the presence of pre‐cultured cells, as implants containing pre‐cultured regenerative cells and abundant cartilage‐like matrix at the time of implantation did not outperform cell‐free implants with the same biomaterial composition and architecture. This observation is of great fundamental, as well as translational importance and supports the hypothesis that functional mimicking of the collagen architecture in the implants may be pivotal for optimal functionality and tissue restoration in vivo.

## AUTHOR CONTRIBUTIONS

**Mylène de Ruijter:** Conceptualization (lead); data curation (lead); formal analysis (lead); investigation (lead); methodology (lead); project administration (lead); resources (lead); validation (lead); visualization (lead); writing – original draft (lead); writing – review and editing (lead). **Paweena Diloksumpan:** Conceptualization (supporting); formal analysis (supporting); methodology (supporting); project administration (supporting); validation (supporting); visualization (supporting); writing – original draft (supporting). **Inge Dokter:** Investigation (supporting); methodology (supporting); validation (supporting). **Harold Brommer:** Investigation (supporting); methodology (supporting). **Ineke H. Smit:** Investigation (supporting); methodology (supporting). **Riccardo Levato:** Conceptualization (supporting); investigation (supporting); methodology (supporting); supervision (supporting); writing – original draft (supporting). **P. René van Weeren:** Conceptualization (supporting); funding acquisition (equal); methodology (supporting); resources (equal); supervision (supporting); writing – original draft (supporting); writing – review and editing (supporting). **Miguel Castilho:** Conceptualization (supporting); data curation (supporting); investigation (supporting); methodology (supporting); project administration (supporting); supervision (equal); writing – original draft (supporting); writing – review and editing (supporting). **Jos Malda:** Conceptualization (equal); funding acquisition (lead); project administration (supporting); resources (lead); supervision (equal); visualization (equal); writing – original draft (equal); writing – review and editing (equal).

## CONFLICT OF INTEREST STATEMENT

The authors declare no conflicts of interest.

### PEER REVIEW

The peer review history for this article is available at https://www.webofscience.com/api/gateway/wos/peer-review/10.1002/btm2.10614.

## Supporting information


**Data S1.** Supporting Information.

## Data Availability

The data that support the findings of this study are available from the corresponding author upon reasonable request.
