# Supplementary material for: Orthotopic equine study confirms the pivotal importance of structural reinforcement over the pre‐culture of cartilage implants
Source: Bioeng Transl Med. 2023 Oct 19;9(1):e10614. doi: 10.1002/btm2.10614 (PMC10771555; doi:10.1002/btm2.10614)
Supplement: Supplementary file 1 — Data S1. Supporting Information. [file BTM2-9-e10614-s001.docx]

**Supplementary data**

**Supplementary figures**


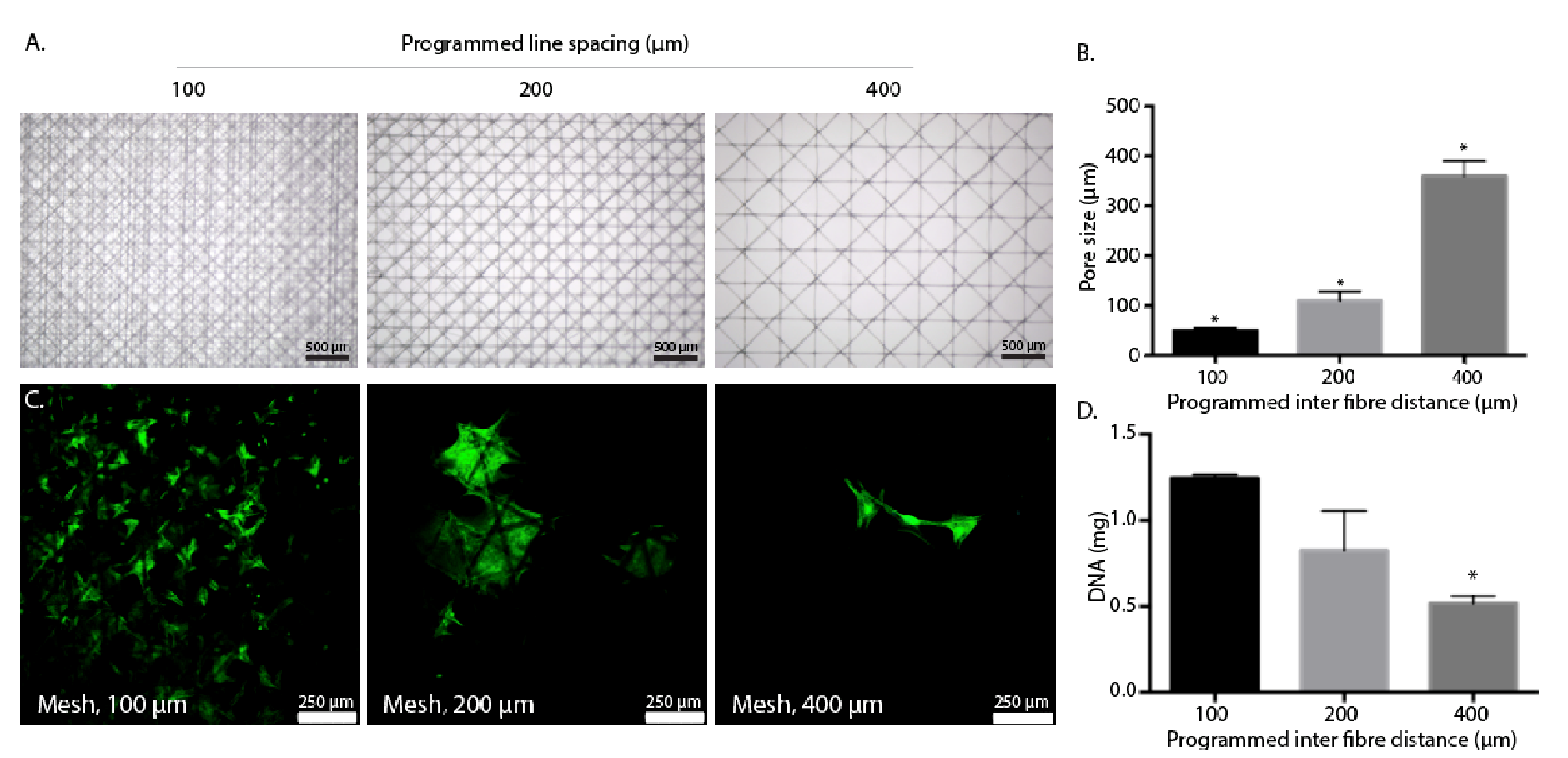


**Supplementary Figure 1.** **Optimization of the superficial tangential MEW reinforcing layer for cell infiltration.** A) Top view images of superficial tangential layers with differently programmed inter-fibre distances. B) Quantification of actual average pore size as a result of programmed inter-fibre distance. C) Fluorescently labelled ACPCs entrapped in meshes with different inter-fibre distances. (LIVE/DEAD staining) D) Quantification of DNA in the superficial layers with different inter-fibre distances. Error bars represent standard deviation * = p < 0.05, one-way ANOVA, post hoc Bonferroni (n = 3).


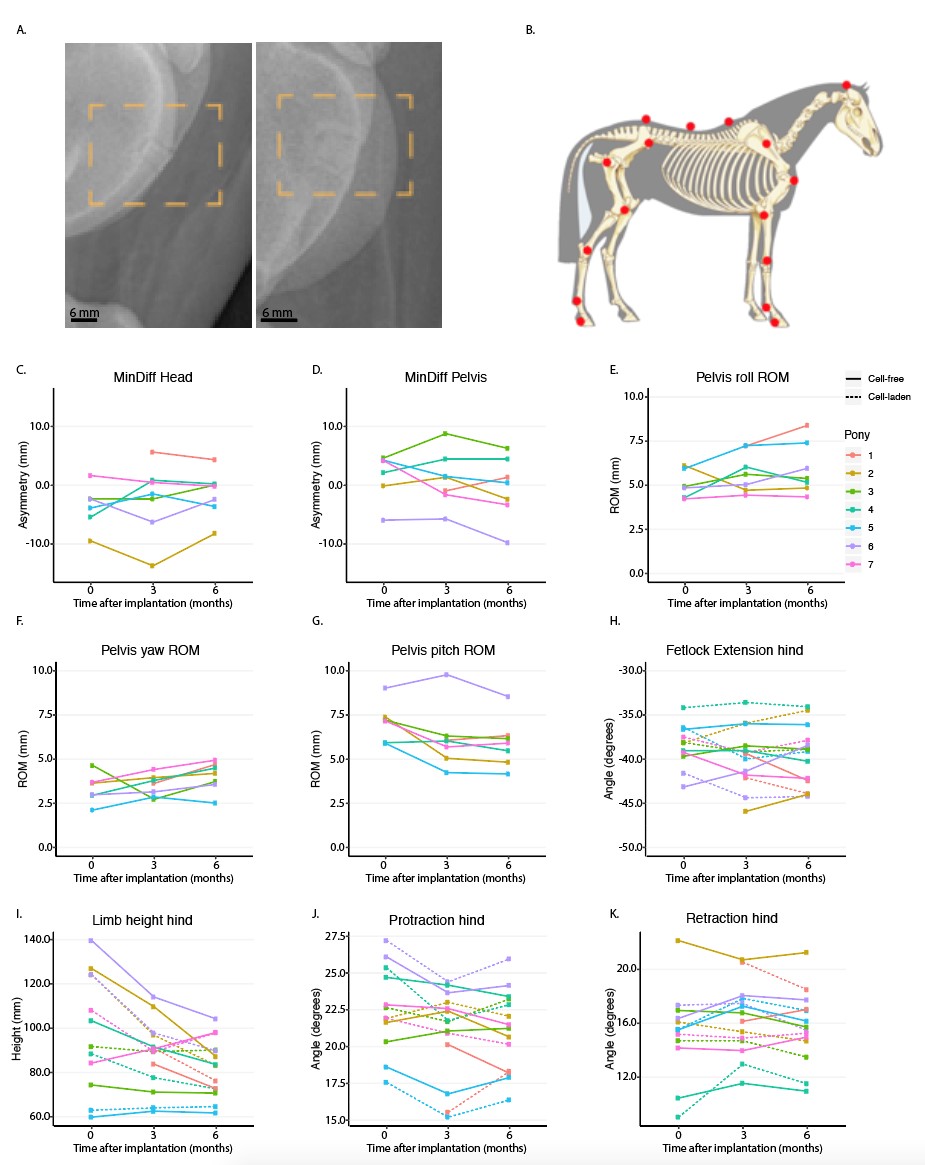


**Supplementary Figure 2. *In vivo* evaluation of osteochondral.** A) Representative X-rays during the implantation period. B) Schematic representation of marker-locations based on anatomical landmarks for gait analysis. C-K) Gait analysis after 0, 3, and 6 months. Legend in E applicable for all graphs. C,D) Symmetry data of the head and pelvis. E) Pelvis roll range of motion (ROM). F) Pelvis Yaw range of motion. G) Pelvis pitch range of motion. H) Fetlock extension hind. I) Limb height hind. J) Protraction hind. K) Retraction hind. T0 =

Prior to implantation. (n = 8).


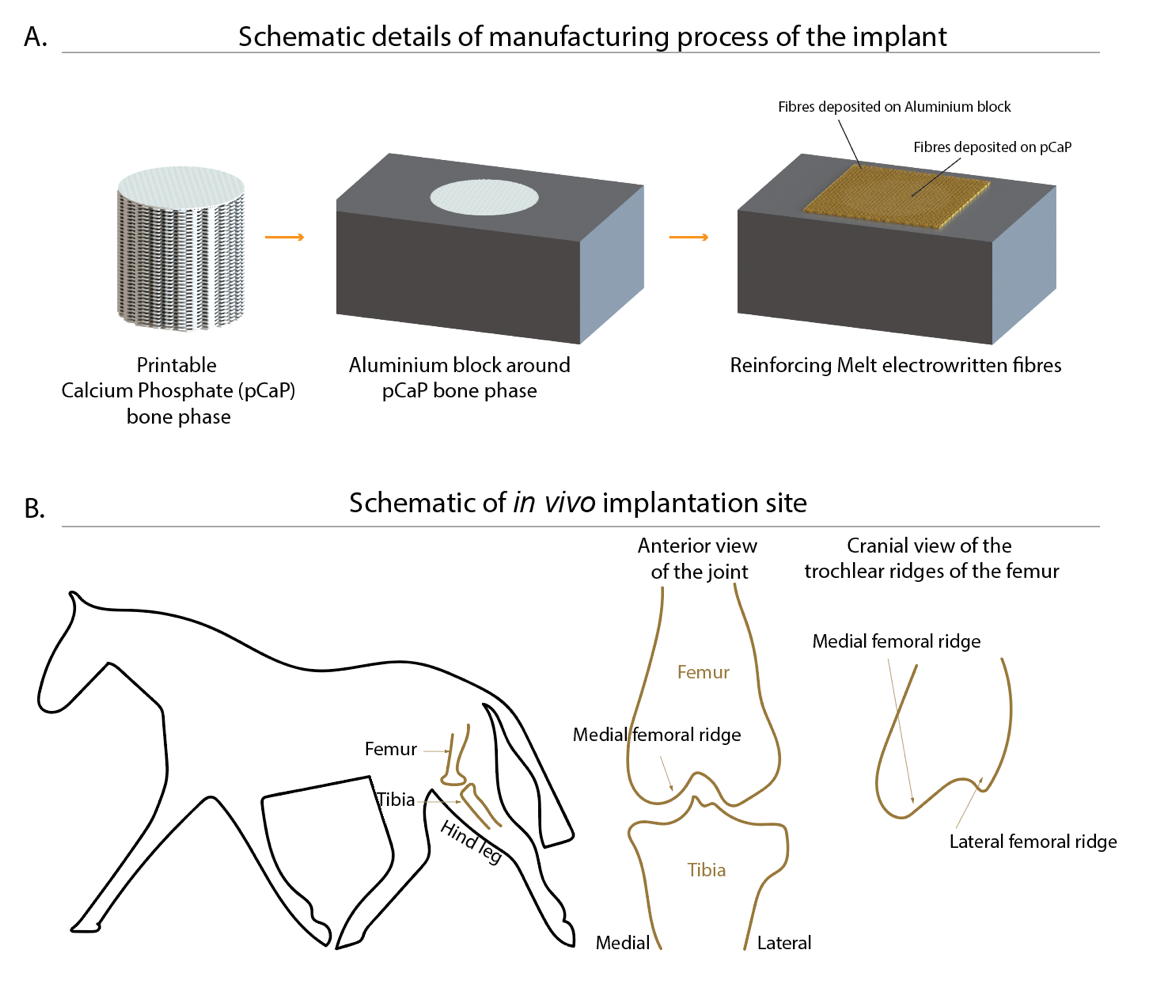


**Supplementary Figure 3. Schematic on manufacturing process and implantation site.** A) Implant manufacturing to prevent piling up of the microfibres at the edges of the implant. Aluminium block is used around the pCaP bone phase to achieve less piling up of the fibres. B) Schematic to illustrate the location of the *in vivo* implantation.


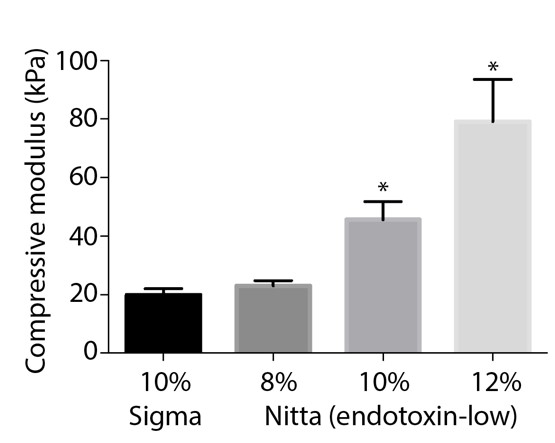


**Supplementary Figure 4. Comparing the compressive modulus of endotoxin-low (Nitta) gelatin-based gelMA with frequently used Sigma gelatin-based gelMA.** 8% endotoxinlow gelMA shows comparative compressive modulus with 10% Sigma gelatin. Error bars represent standard deviation. * = p < 0.05, one-way ANOVA, post hoc Bonferroni (n=5).


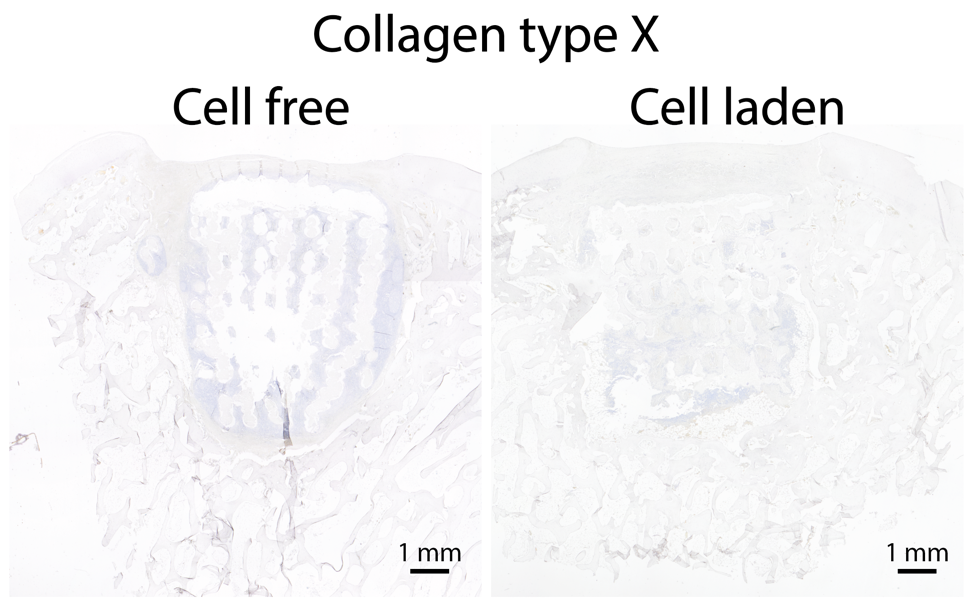


**Supplementary Figure 5. Representative images of collagen type X staining to analyze hypertrophic differentiation after 6 months of in vivo implantation.**  The cell free and cell laden groups both stain negative for collagen type X staining.

**Supplementary methods**

Immunohistochemistry was performed to visualize collagen type X deposition. First, sections were deparaffinized, washed in PBS, blocked with 0.3%H_2_O_2_ solution (in PBS), and washed with 0.1% PBS-Tween. Antigen retrieval was done with pepsin (1 mg pepsin, Sigma P7012, in 1 ml of 0.5M acetic acid) for 2 hours at 37°C and Hyaluronidase (10mg/ml, Sigma H2126) in PBS) for 30 minutes at 37°C. Sections were washed with PBS-Tween, blocked with 5% PBS/BSA for 30 minutes at room temperature. Sections were then incubated with the primary antibody (Collagen type X, Mouse monoclonal Antibody, Quartett, 2031501005) at a 1:200 dilution in PBS/BSA over night at 4°C. IgG was used as negative control staining. Sections were washed with PBS-Tween and incubated with the secondary antibody (Anti Mouse IgG, biotynilated, GE Healthcare, RPN1001V) at a dilution of 1:200 in PBS/BSA 5%) for 60 minutes at room temperature and washed with PBS-Tween again. Subsequently, 3,3-diaminobenzidine-horseradish peroxidase (DAB, Sigma Aldrich, USA) was used to visualize the staining. After staining the cell nuclei with haematoxylin, pictures of histologically stained sections were made with a light microscope (Olympus BX51, The Netherlands).
